# Comparison of diurnal variation, anatomical location, and biological sex within spontaneous and driven dynamic cerebral autoregulation measures

**DOI:** 10.14814/phy2.14458

**Published:** 2020-06-14

**Authors:** Joel S. Burma, Paige Copeland, Alannah Macaulay, Omeet Khatra, Jonathan D. Smirl

**Affiliations:** ^1^ Concussion Research Laboratory Faculty of Health and Exercise Science University of British Columbia Kelowna BC Canada; ^2^ Sport Injury Prevention Research Centre Faculty of Kinesiology University of Calgary Calgary AB Canada; ^3^ Human Performance Laboratory Faculty of Kinesiology University of Calgary Calgary AB Canada; ^4^ Hotchkiss Brain Institute University of Calgary Calgary AB Canada; ^5^ Integrated Concussion Research Program University of Calgary Calgary AB Canada; ^6^ Faculty of Medicine University of British Columbia Vancouver BC Canada; ^7^ Alberta Children's Hospital Research Institute University of Calgary Calgary AB Canada; ^8^ Libin Cardiovascular Institute of Alberta University of Calgary Calgary AB Canada

**Keywords:** cerebral autoregulation, diurnal variation, sex differences, spontaneous, squat‐stand maneuvers

## Abstract

Presently, the literature describing the influence of diurnal variation on dynamic cerebral autoregulation (dCA) metrics is sparse. Additionally, there is little data with respect to dCA comparisons between anterior/posterior circulation beds and biological sexes using squat‐stand maneuvers. Eight male and eight female participants (*n* = 16) performed 5 min of spontaneous upright rest and squat‐stand maneuvers at 0.05 and 0.10 Hz across seven time points throughout the day. All testing sessions commenced at 8:00 a.m. each day and dCA parameters were quantified across the cardiac cycle (diastole, mean, and systole) using transcranial Doppler ultrasound to insonate cerebral blood velocity within the middle and posterior cerebral arteries (MCA, PCA). No cardiac cycle alternations were seen spontaneous (all *p* > .207) while a trend was noted in some driven (all *p* > .051) dCA metrics. Driven dCA produced much lower coefficient of variances (all <21%) compared with spontaneous (all <58%). Moreover, no sex differences were found within driven metrics (all *p* > .096). Between vessels, PCA absolute gain was reduced within all spontaneous and driven measures (all *p* < .014) whereas coherence, phase, and normalized gain were unchanged (all *p* > .099). There appears to be little influence of diurnal variation on dCA measures across the day (8:00 a.m. to 6:00 p.m.). Absolute gain was blunted in the PCA relative to the MCA and consistent with previous literature, driven methods demonstrated vastly improved reproducibility metrics compared to spontaneous methods. Finally, no dCA differences were found between biological sexes, demonstrating that males and females regulate in a harmonious manner, when females are tested within the early follicular phase of the menstrual cycle.

## Graphical abstract

Driven dynamic dynamic cerebral autoregulation (dCA) metrics elicited more robust measures compared to spontaneous metrics across the day (8:00–18:00). Additionally, both the middle and posterior cerebral arteries regulated similarly across the day, as well did both males and female participants. Therefore, in the future when feasible, squat‐stand maneuvers should be used to assess dCA in participants, which can be performed in both vessels, across the cardiac cycle and in both biological sexes, as there is minimal influence of diurnal variation across the day within these measures.

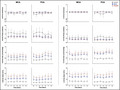

## 1 INTRODUCTION

Cerebral autoregulation refers to the brain's ability to regulate cerebral blood velocity (CBV) and cerebral perfusion, somewhat independent to the rest of the body (Aaslid, Lindegaard, Sorteberg, & Nornes, 1989; Lassen, 1959; Willie, Tzeng, Fisher, & Ainslie, 2014). The notion of cerebral autoregulation has evolved since it was first proposed in 1959 by Lassen and now is considered a spectrum dominated by two domains: dynamic and static (Aaslid et al., 1989; Lassen, 1959). Dynamic cerebral autoregulation (dCA) alludes to the frequency range where autoregulation is known to be present (0.02–0.20 Hz), whereas static cerebral autoregulation most often refers to a process where steady‐state measures of blood pressure are taken pre/post a system perturbation (Claassen, Levine, & Zhang, 2009; Zhang, Zuckerman, Giller, & Levine, 1998) (Claassen et al., 2009; Zhang et al., 1998).

There is currently an ongoing debate regarding the best way to quantify dCA, where Tzeng and Panerai (2018) suggest dCA should be indexed using spontaneous measures using transfer function analysis (TFA) to assess the linearity between fluctuations in blood pressure and CBV. These authors argue this approach is safer for clinical populations as rapid elevations in blood pressure are not induced, as well it does not challenge the autonomic or respiratory systems (Tzeng & Panerai, 2018). Nonetheless, a major drawback of this method is the minimal level of blood pressure variability it elicits, which results in lower TFA coherence levels and diminished linearity within the cerebral pressure–flow relationship (Smirl, Hoffman, Tzeng, Hansen, & Ainslie, 2015). Moreover, as there is potential for other physiological parameters (i.e., respiration rates, hormonal fluctuations, neural activation, etc.) to influence the TFA metrics, the linearity between blood pressure and CBV is confounded (Smirl et al., 2015). Contrarily, Simpson and Claassen (2018) opposed the aforementioned view of Tzeng and Panerai (2018) suggesting induced oscillations should be used over spontaneous, as the former are more physiological and clinically applicable for everyday challenges, as various common daily activities result in massive swings of blood pressure (i.e., walking upstairs, bending over, defecating, etc.).

Moreover, Smirl et al. (2015) conducted a study examining the between‐day reproducibility comparisons of driven (squat‐stand maneuvers and oscillatory lower body negative pressure) and spontaneous measures within young healthy adults (25 ± 3 years) and older (66 ± 4 years) individuals. It was found that driven oscillations were capable of producing coherence values in excess of 0.99 compared to spontaneous coherence values ranging from 0.53 to 0.88, and therefore squat‐stands are considered to be the gold standard for assessing the linear aspect of the dCA relationship between systemic blood pressure and CBV (Smirl et al., 2015). Ultimately, squat‐stand maneuvers enhance the signal‐to‐noise ratio within the cerebral pressure–flow relationship, which eradicates the likelihood the dCA metrics are impacted by physiological “noise” within the measurement which often can play a significant role when spontaneous blood pressure oscillations are employed to assess dCA metrics. Additionally, within the aforementioned investigation (Smirl et al., 2015), these authors revealed squat‐stand measures reduced the within‐subject between‐day coefficient of variance (CoV) within all variables of interest ranging from 0.1% to 19.4%. In contrast, both the upright spontaneous position (range: 8.4%–35.6%) and oscillatory lower‐body negative pressure technique (range: 2.6%–51.3%) elicited a large variation within these same variables. Finally, Smirl, Wright, Ainslie, Tzeng, and van Donkelaar (2018) have demonstrated squat‐stand maneuvers are highly reliable and capable of producing coherence values >0.90 across all phases of the cardiac cycle (i.e., diastole, mean, and systole), showing the utility of this methodology to quantify dCA. Nevertheless, there is an absence of literature examining the: within‐day reproducibility of these measures; alterations across the cardiac cycle in both spontaneous and driven oscillations; and the influence of diurnal variation on these parameters. Moreover, there have only been limited studies examining the cerebral pressure–flow relationship at either diastole and/or systole, which has shown to reveal more information than mean independently at rest (Smirl et al., 2018), during and following exercise (Ogoh et al., 2005, 2007), and in concussion (Wright, Smirl, Bryk, & van Donkelaar, 2018). In addition, there are only a smattering of investigations that have used squat‐stand maneuvers to examine dCA differences between anatomical circulations or between biological sexes (Favre & Serrador, 2019; Labrecque et al., 2019).

Therefore, the primary purpose of this investigation was to compare the within‐day reproducibility and potential influence of diurnal variation on both spontaneous and driven dCA measures across the cardiac cycle. Furthermore, this study aimed to discern potential biological sex differences in dCA metrics and examine if any dCA disparities exist between circulatory beds using squat‐stand maneuvers as this methodology has shown to be highly reliable between days compared to other methods used to quantify dCA (Burma et al., 2020). It was hypothesized that: (a) diurnal variation would influence both spontaneous and driven dCA metrics consistent with previous research (Cummings, Swart, & Ainslie, 2007); (b) driven blood pressure oscillations would elicit greater within‐day reproducibility than spontaneous measures, represented by a lower CoV within all dCA variables of interest (Smirl et al., 2015); (c) the middle (MCA) and posterior cerebral arteries (PCA) would regulate differently (Haubrich, Wendt, Diehl, & Klötzsch, 2004); and (d) there would be differences in autoregulatory capacity between biological sexes (Favre & Serrador, 2019; Labrecque et al., 2019).

## 2 MATERIALS AND METHODS

### 2.1 Study design

A written informed consent was collected before engagement in this study, which was approved by the University of British Columbia clinical ethics review board (H16‐00507). Eight females and eight males (*n* = 16) were recruited from the university setting to partake in this investigation. All participants were in good health with no history of neurological, cerebrovascular, cardiorespiratory, or musculoskeletal complications. Participants performed the standing (spontaneous) and squat‐stand (driven) protocol at seven time points throughout the day. To ensure the comparison between individuals were similar, all testing was initiated at 8:00 a.m. each day, and successive protocols were executed at 9:00 a.m., 10:00 a.m., 11:00 a.m., 1:00 p.m., 3:00 p.m., and 5:00 p.m. All participants remained within the testing location to ensure they remained at rest and were instructed to sit quietly for 5 min before each testing session, to minimize the likelihood any covariates (exercise, eating, postural alterations) played a significant role in influencing the data (Burma et al., 2020). Furthermore, female participants were tested between days 3 and 7 of their menstrual cycle (early follicular phase), as during this time frame, hormonal levels are known to be more stable (Abidi et al., 2017; Boivin & Shechter, 2010; Krejza et al., 2003). All testing protocols were thoroughly explained and demonstrated by the examiner prior to data collections to ensure participants performed the procedures in a consistent manner. All participants abstained from caffeine, and alcohol for a minimum of 12 hr, before engagement within this study, similar to previous research in this area (Ainslie, Barach, et al., 2007; Ainslie, Hamlin, Hellemans, Rasmussen, & Ogoh, 2008; Smirl et al., 2015; Smirl, Hoffman, Tzeng, Hansen, & Ainslie, 2016). Exercise was restricted for a minimum of 6 hr prior to data collection, consistent with the time‐course recovery of dCA metrics following both moderate‐ and high‐intensity exercise (Burma et al., 2020). Diet was controlled for across the day as participants consumed two meal replacement drinks (Vanilla Nutrition Shake, Kirkland Signature; 210 calories each) between 11:30 a.m. and 1:00 p.m. and two Gatorades across the day (Gatorade Perform, PepsiCo; 150 calories each). Lastly, participants had access to water and were able to use the washroom as they required.

This study was a subsection of a larger study examining the postexercise effects moderate‐intensity continuous training (MICT) and high‐intensity interval training (HIIT) have on cardiovascular (cardiac baroreceptor sensitivity and heart rate variability) and cerebrovascular function (neurovascular coupling, cerebrovascular reactivity, and dCA; Burma et al., 2020). The current study examines the effect of diurnal variation on dCA measures, determines if differences exist between biological sexes, and the extent how dCA is congruently regulated within anterior and posterior cerebral circulatory beds. It is important to note that the control data from eight of the participants, who completed the overall study including the MICT and HIIT interventions, were used within this investigation (50%). Therefore, half of the data are from individuals who performed the control intervention independently.

### 2.2 Instrumentation

Heart rate was obtained through the use of a three‐lead electrocardiogram (ECG). Transcranial Doppler ultrasound was employed to insonate and quantify the right MCA blood velocity and the left PCA blood velocity in real‐time by placing 2‐MHz ultrasound probes (Spencer Technologies) over the temporal acoustic windows. Once vessels were insonated and identified, they were confirmed with a visual task and a carotid artery compression challenge (Willie et al., 2011). After confirmation, the probes were locked into place using a fitted head‐frame (Spencer Technologies). Beat‐to‐beat blood pressure was recorded using finger photoplethysmography and was corrected to the height of the heart (Finometer PRO; Finapres Medical Systems; Omboni et al., 1993; Sammons et al., 2007). End‐tidal partial pressure of carbon dioxide (P_ET_CO_2_) was measured with an online gas analyzer (ML206; AD Instruments) and was calibrated with known gas concentrations prior to data collection. Data were sampled at a frequency of 1,000 Hz (PowerLab 8/30 ML880; AD instruments) time‐locked, and stored for offline analysis with commercially available software (LabChart version 7.1; AD Instruments).

### 2.3 Experimental protocols

**FIGURE 1:**
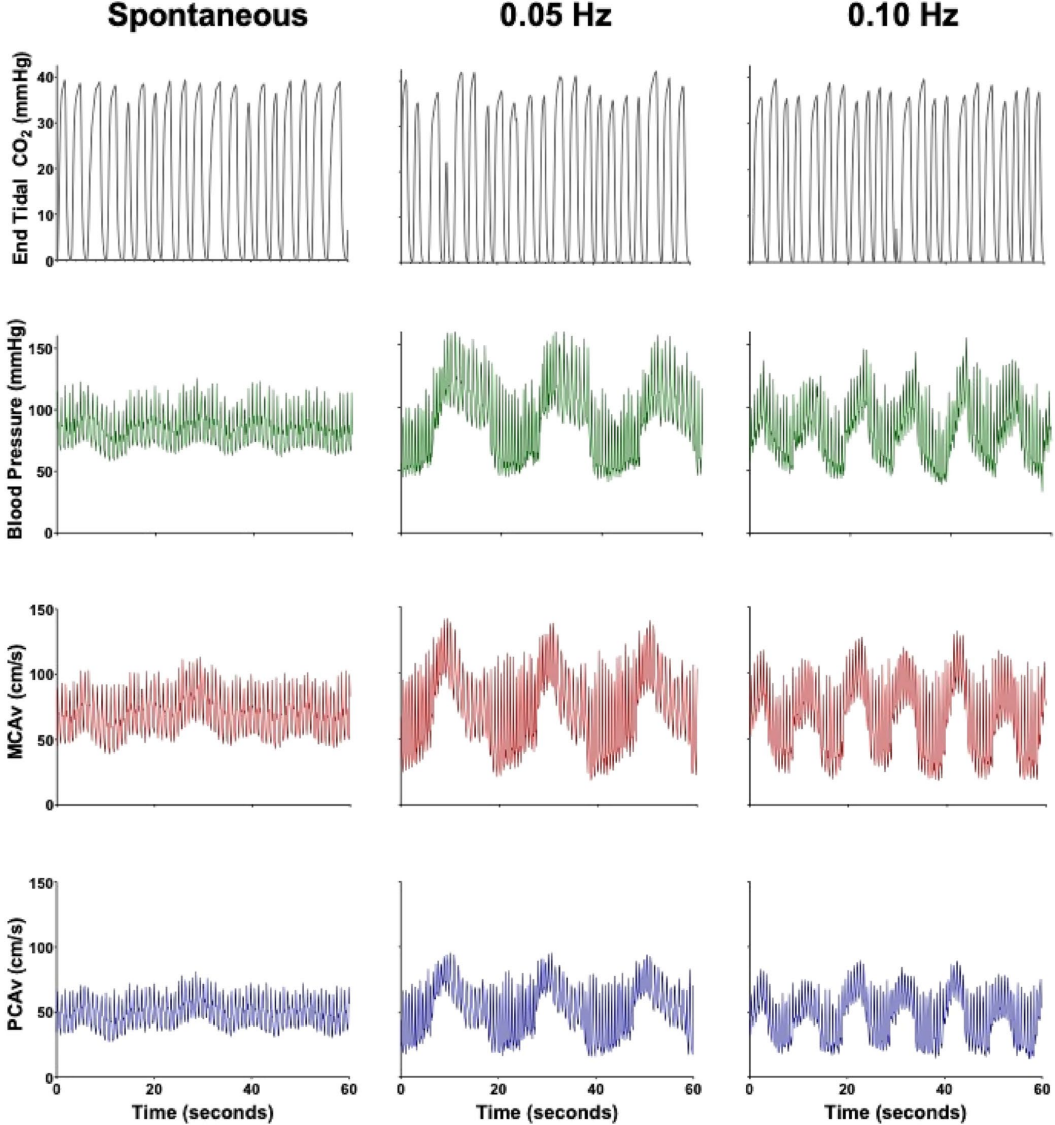
Representative trace of an individual performing 5 min of spontaneous data and 5 min of squat‐stand maneuvers at 0.05 and 0.10 Hz. The measurements displayed are end‐tidal partial pressure of carbon dioxide, blood pressure, middle cerebral artery blood velocity (MCAv), and posterior cerebral artery blood velocity (PCAv). Note: The 30–50 mmHg swing in blood pressure and the associated changes to MCAv and PCAv can be seen within the squat‐stand maneuvers at both frequencies

Participants performed 5 min of quiet‐standing, which was used to quantify dCA under the influence of spontaneous oscillations in blood pressure. During this time, participants stood still for 5 min while facing forwards, eyes open and were instructed to remain motionless. Upon completion of the quiet‐stance period, the participants performed two sets of squat‐stand maneuvers, which have been demonstrated to be the *gold standard* for assessing the linearity between blood pressure and CBV (Smirl et al., 2015). To perform the squat‐stand maneuvers, participants began in an upright position and proceeded to squat down until a ~90° angle was created between the hamstring and calf along the back of their leg. The squats were performed for 5 min at two separate frequencies of 0.05 Hz and 0.10 Hz, which were paced with a metronome. For the 0.05‐Hz squat‐stand maneuvers, participants performed alternating 10‐s squat and 10‐s standing periods, while for the 0.10‐Hz maneuvers participants performed with 5‐s cycles. These frequencies were selected as they are within the very low frequency (VLF) range (0.02–0.07 Hz) and low frequency (LF; 0.07–0.20 Hz) frequency bands, which are consistent with the most active aspect of dCA (<0.20 Hz; Claassen et al., 2009). Furthermore, these frequencies have been proposed to reveal information about cerebrovascular regulation being altered due to either metabolic, myogenic, or endothelial influences (0.05 Hz) and sympathetic innervation (0.10 Hz; Hamner & Tan, 2014; Zhang et al., 1998). A representative trace of an individual performing the spontaneous and driven blood pressure oscillations and the associated physiological parameters is displayed in Figure 1.

### 2.4 Data processing

Diastolic, mean, and systolic measures of blood pressure and CBV were derived from the R–R intervals from the ECG. The P_ET_CO_2_ levels were indexed using breath‐to‐breath peak of expired carbon dioxide levels and all signals were visually inspected for artifacts. Data were processed and analyzed using commercially available software (Ensemble) which adheres to the standards and rigors established in the Cerebral Autoregulation Research Network (CARNet) white paper (Claassen, Meel‐van den Abeelen, Simpson, & Panerai, 2015). Point‐estimates were taken from the driven frequencies of interest (0.05 and 0.10 Hz), whereas the band averages were taken in the VLF (0.02–0.07 Hz) and LF (0.07–0.20 Hz) ranges within the spontaneous data, consistent with prior research (Smirl, Tzeng, Monteleone, & Ainslie, 2014; Smirl, Haykowsky, et al., 2014; Smirl, Lucas, et al., 2014; Smirl et al., 2015, 2016, 2018; Kostoglou et al., 2016; Wright et al., 2018, 2018a, 2018b). The dCA outcomes variables of interest within this investigation include coherence, phase, absolute gain, and normalized gain. Coherence describes the linearity between mean arterial pressure (input) and CBV (output), where a value of 0.0 describes an absence of a relationship and 1.0 characterizes a completely linear relationship (Zhang et al., 1998). Phase portrays the timing offset between the aforementioned variables, whereas gain refers to the amplitude modulation between variables (Zhang et al., 1998).

### 2.5 Statistical analyses

#### 2.5.1 Sample size calculation

A sample size calculation was determined from the squat‐stand methodological paper (*n* = 10), which congruently compared spontaneous to driven dCA methodology at two time points rather than seven (Smirl et al., 2015). Calculations were performed for each of the TFA variables at both frequencies with two‐tails, at an alpha of 0.05, and a power of 0.80, using the mean and standard deviations between subjects the first testing day, within the aforementioned study (Smirl et al., 2015). These values were averaged, and it was determined that a sample size of eight individuals was needed for the current investigation. Nonetheless, for the diurnal variation and vessel difference comparisons, the data were compiled meaning 16 subjects were in each group, giving an adequately powered study. Moreover, this sample size is in direct comparison, with a plethora of previous research examining dCA, that had sample sizes of seven (Ainslie, Hamlin, et al., 2008; Kim et al., 2007; Ogoh et al., 2005), eight (Bailey et al., 2011; Claassen et al., 2009; Serrador & Freeman, 2017; Smirl, Haykowsky, et al., 2014), nine (Falvo, Lindheimer, & Serrador, 2018; Ogoh, Nakahara, Ainslie, & Miyamoto, 2010; Ogoh et al., 2016; Smirl, Haykowsky, et al., 2014; Smirl et al., 2016; Tsukamoto et al., 2019), or 10 participants (Aaslid et al., 1989; Ainslie, Celi, McGrattan, Peebles, & Ogoh, 2008; Smirl, Haykowsky, et al., 2014; Smirl et al., 2015, 2016).

#### 2.5.2 Diurnal variation

Statistical analyses were performed using SPSS version 25.0. One (dCA metric) by seven (time: baseline, 0, 1, 2, 4, 6, and 8 hr follow‐ups) within‐subjects repeated‐measures ANOVA were performed for each TFA measure of interest (coherence, phase, absolute gain, and normalized gain). A priori Bonferroni post hoc corrected comparisons for simple effects were performed between the follow‐up time points to establish if any time point was different from the others. Data are presented as mean ± *SD*. Significance was set a priori at *p* < .05.

#### 2.5.3 Sex and vessel differences

Independent *t* tests were run for all dCA metrics (coherence, phase, absolute gain, and normalized gain) to determine if any measures were different between biological sexes or vessels. Each individual's data were collapsed across the seven time points in order to provide an averaged within‐subject estimate of dCA. Bonferroni corrections were applied for comparisons performed across the cardiac cycle (diastole, mean, and systole). Data are presented as mean ± *SD*. Significance was set a priori at *p* < .05.

## 3 RESULTS

### 3.1 Participant demographics

Female participants had an average age of 24 ± 3 years, height of 168 ± 7 cm, weight of 61 ± 6 kg, body mass index (BMI) of 22 ± 2 kg/cm^2^, resting standing blood pressure of 92 ± 6 mmHg, and resting standing heart rate of 85 ± 6 bpm (Table 1). Male participants had an average age of 25 ± 5 years, height of 181 ± 8 cm, weight of 84 ± 14 kg, body mass index of 25 ± 4 kg/cm^2^, resting standing blood pressure of 91 ± 9 mmHg, and resting standing heart rate of 80 ± 7 bpm (Table 1).

**TABLE 1:** Participant demographics

Metric	Male (n = 8)	Female (n = 8)	Total (n = 16)
Age (years)	25 ± 5	24 ± 3	25 ± 4
Height (cm)	181 ± 8	168 ± 7	174 ± 10
Weight (kg)	84 ± 14	61 ± 6	72 ± 16
BMI (kg/cm^2^)	25 ± 4	22 ± 2	24 ± 4
Resting standing mean arterial pressure (mmHg)	91 ± 9	92 ± 5	91 ± 7
Resting standing heart rate (bpm)	80 ± 7	85 ± 6	82 ± 7

Note

Values are mean ± *SD*. It is important to note that the average mean arterial pressure and heart rate were taken while standing and thus participants would have a slightly raised sympathetic contribution. Therefore, the heart rates would be elevated from what would been seen while sitting.

Abbreviations: BMI, body mass index; bpm, beats per minute; cm, centimetres, kg, kilograms, mmHg, millimetres of mercury.

### 3.2 Cardiovascular and cerebrovascular metrics across the day

At all seven time points across the day, all cardio‐ and cerebrovascular parameters were consistent during the 0.05 Hz squat‐stand maneuvers (P_ET_CO_2_: all *p* > .565; MCA blood velocity: all *p* > .520; PCA blood velocity: all *p* > .111; mean arterial pressure: all *p* > .544; heart rate: all *p* > .229; Table 2). Likewise, all of the aforementioned variables were congruent across all time points at 0.10 Hz (P_ET_CO_2_: all *p* > .140; MCA blood velocity: all *p* > .358; PCA blood velocity: all *p* > .186; mean arterial pressure: all *p* > .166; heart rate: all *p* > .334; Table 2).

**TABLE 2:** Cardiovascular and cerebrovascular variables during spontaneous and squat‐stand maneuvers across each time point at 0.05 and 0.10 Hz

	08:00	9:00	10:00	11:00	13:00	15:00	17:00
Spontaneous							
P_ET_CO_2_ (mmHg)	38 ± 3	38 ± 3	37 ± 3	37 ± 3	38 ± 3	38 ± 3	37 ± 3
MCAbv (cm/s)	60 ± 6	60 ± 6	59 ± 5	59 ± 6	60 ± 8	59 ± 6	59 ± 5
PCAbv (cm/s)	34 ± 5	35 ± 6	36 ± 6	36 ± 6	36 ± 7	35 ± 5	35 ± 5
MAP (mmHg)	89 ± 7	94 ± 11	95 ± 7	93 ± 8	93 ± 7	93 ± 9	97 ± 12
HR (bpm)	83 ± 10	84 ± 6	81 ± 7	81 ± 8	89 ± 11	81 ± 13	83 ± 12
0.05 Hz							
P_ET_CO_2_ (mmHg)	39 ± 3	39 ± 3	38 ± 3	37 ± 3	39 ± 2	39 ± 2	38 ± 4
MCAbv (cm/s)	61 ± 7	60 ± 8	59 ± 7	59 ± 8	61 ± 9	59 ± 8	59 ± 7
PCAbv (cm/s)	33 ± 7	34 ± 7	34 ± 7	35 ± 7	35 ± 8	34 ± 6	35 ± 7
MAP (mmHg)	93 ± 8	99 ± 12	98 ± 9	98 ± 11	95 ± 9	97 ± 9	98 ± 13
HR (bpm)	89 ± 12	88 ± 13	89 ± 13	91 ± 13	95 ± 11	91 ± 15	92 ± 12
0.10 Hz							
P_ET_CO_2_ (mmHg)	40 ± 3	39 ± 3	39 ± 3	39 ± 3	40 ± 3	40 ± 3	39 ± 3
MCAbv (cm/s)	62 ± 7	61 ± 7	61 ± 8	61 ± 7	62 ± 9	60 ± 7	60 ± 7
PCAbv (cm/s)	34 ± 7	34 ± 7	35 ± 8	35 ± 7	35 ± 8	34 ± 7	35 ± 7
MAP (mmHg)	94 ± 9	99 ± 15	99 ± 7	99 ± 9	96 ± 10	97 ± 12	98 ± 13
HR (bpm)	88 ± 11	87 ± 12	89 ± 13	91 ± 12	94 ± 10	93 ± 13	91 ± 13

Note

Values are means ± *SD*.

Abbreviations: bpm, beats per minute; cm/s, centimeters per second; HR, heart rate; Hz, Hertz; MAP, mean arterial pressure; MCAbv, middle cerebral artery blood velocity; mmHg, millimeters of mercury; PCAbv, posterior cerebral artery blood velocity; P_ET_CO_2_, end‐tidal values of carbon dioxide.

### 3.3 Variations in autoregulation across the day

Between spontaneous and squat‐stand maneuvers at 0.05 Hz, blood pressure power spectrum density was increased an average of 78‐fold in diastole, 86‐fold in mean, and 96‐fold in systole, whereas at 0.10 Hz, it was augmented an average of 58‐fold at diastole, 69‐fold at mean, and 46‐fold at systole. The MCA power spectrum density was enhanced an average of 49‐fold in diastole, 47 in mean, and 46 in systole at 0.05 Hz and 46‐fold in diastole, 41‐fold in mean, and 11‐fold in systole at 0.10 Hz. At 0.05 Hz, the average PCA power spectrum density elevated 31‐fold in diastole, 45‐fold in mean, and 52‐fold in systole, whereas at 0.10Hz, it increased 43‐fold in diastole, 42‐fold in mean, and 11‐fold in systole. These did not differ between sexes in both driven (all *p* > .148) and spontaneous (all *p* > .066) or between the vasculature (all *p* > .197). Furthermore, at 0.05Hz, the blood pressure power spectrum density was congruent between biological sexes at diastole (female: 14,092 ± 3,366 mmHg^2^; male: 17,385 ± 8,155 mmHg^2^), mean (female: 23,436 ± 4,613 mmHg^2^; male: 25,776 ± 12,621 mmHg^2^), and systole (female: 37,307 ± 10,428 mmHg^2^; male: 45,169 ± 29,056 mmHg^2^; all *p > .857*). Likewise, at 0.10 Hz, the blood pressure power spectrum density was similar between biological sexes at diastole (female: 16,466 ± 3,464 mmHg^2^; Male: 16,522 ± 5,608 mmHg^2^), mean (female: 21,973 ± 4,383 mmHg^2^; male: 21,571 ± 7,482 mmHg^2^), and systole (female: 22,761 ± 5,116 mmHg^2^; male: 28,759 ± 9,154 mmHg^2^; all *p* > .384).

**FIGURE 2:**
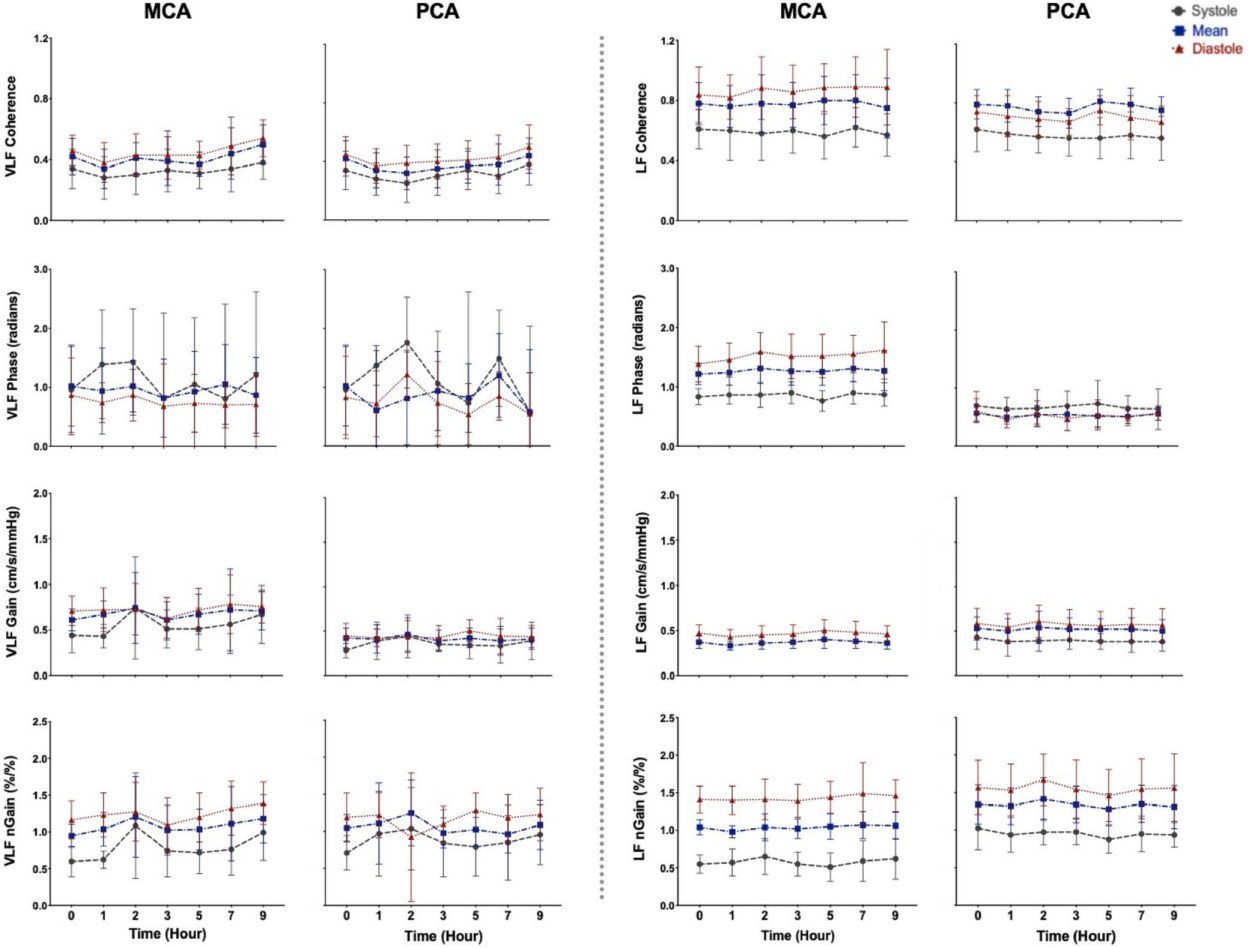
Mean with *SD* (error bars) of coherence, phase, gain, and normalized gain spontaneous values within the middle cerebral artery (MCA) and the posterior cerebral artery (PCA) across the cardiac cycle (*n* = 16). The data were taken as a band average within the very‐low‐frequency (VLF) and low‐frequency (LF) ranges. Systole is shown with gray (dashed) data points, mean is shown with blue (dashed‐dotted) data points, and diastole is shown with red (dotted) data points

**FIGURE 3:**
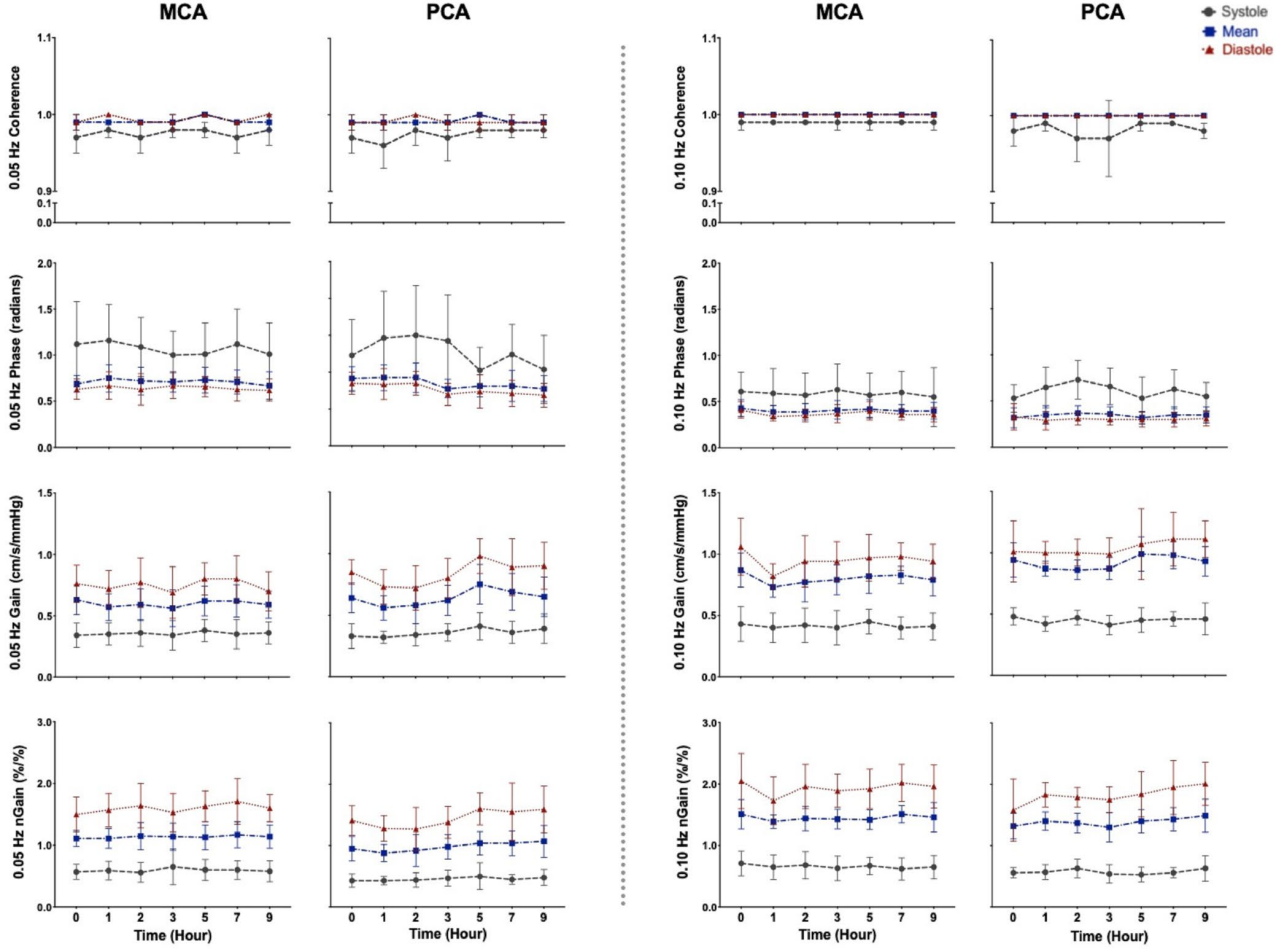
Mean with *SD* (error bars) of coherence, phase, gain, and normalized gain driven values within the middle cerebral artery (MCA) and posterior cerebral artery (PCA) across the cardiac cycle (*n* = 16). Squat‐stand maneuvers were driven at point‐estimates of 0.05 and 0.10 Hz, which fall within the very‐low‐frequency and lowfrequency ranges, respectively. The gray (dashed), blue (dashed‐dotted), and red (dotted) data points represent the systolic, mean, and diastolic components of the cardiac cycle, respectively

**FIGURE 4:**
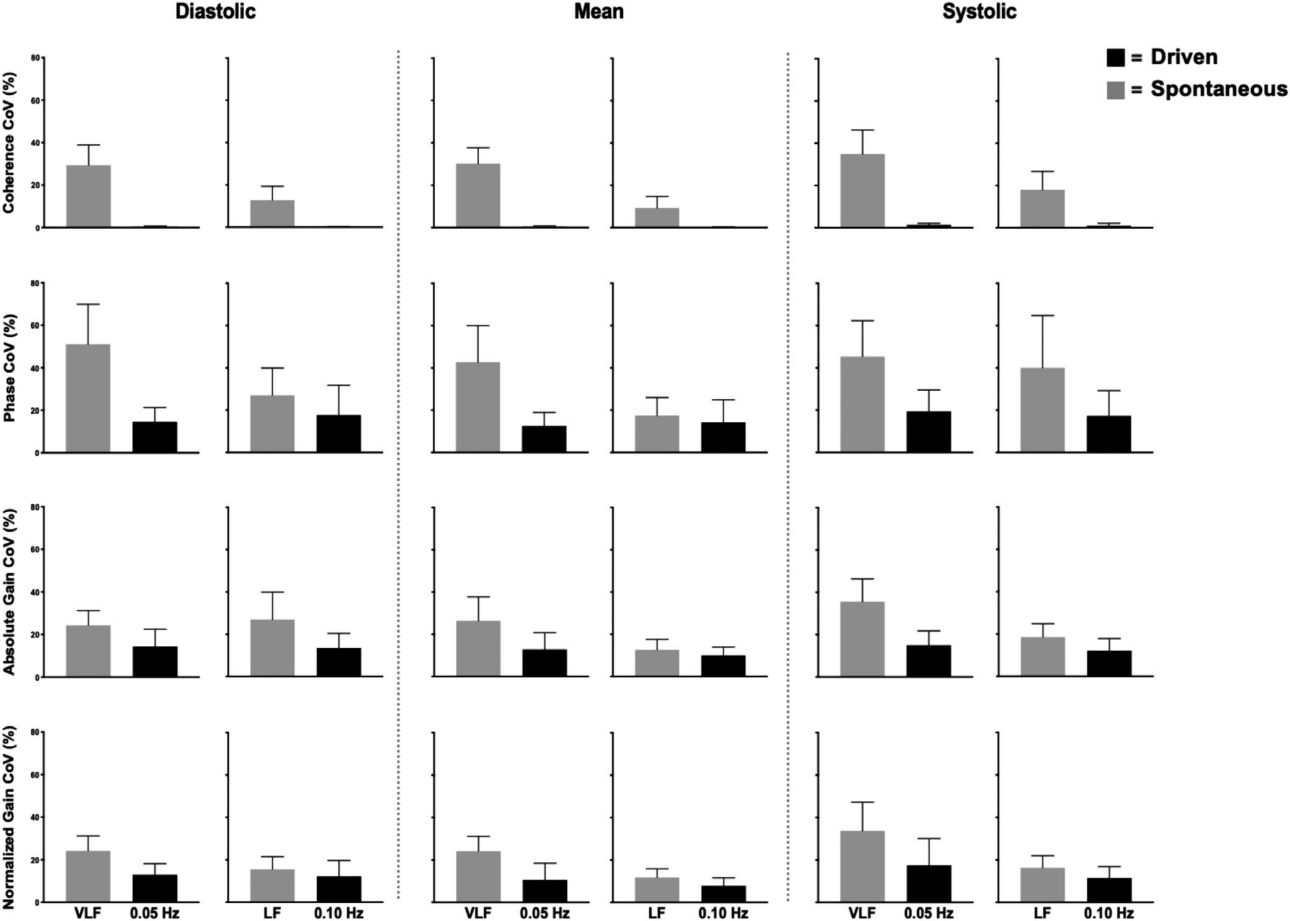
Mean with *SD* (error bars) of the coefficient of variance (CoV) across coherence, phase, gain, and normalized gain values within the middle cerebral artery (*n* = 16). Spontaneous measures (gray bars) were collected as band averages within the very low frequency and low frequency, whereas driven measures (black bars) were collected through squat‐stand maneuvers at point‐estimates of 0.05 and 0.10 Hz

**FIGURE 5:**
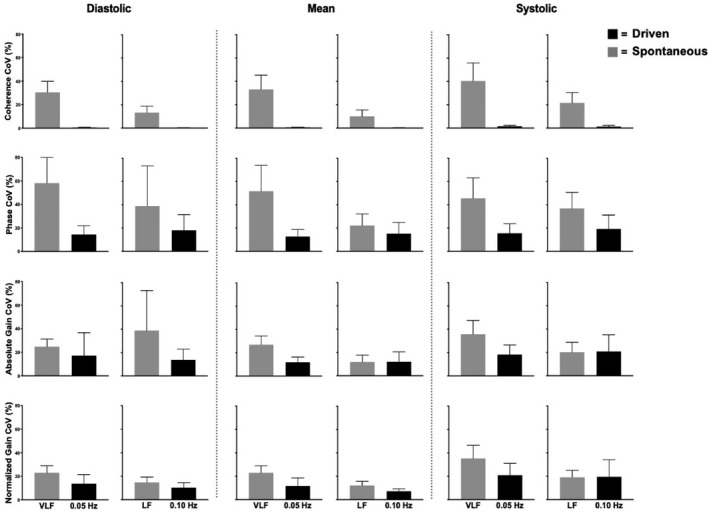
Mean with *SD* (error bars) of the coefficient of variance (CoV) across coherence, phase, gain, and normalized gain values within the posterior cerebral artery (*n* = 16). Spontaneous measures were collected as band averages within the very low frequency (VLF) and low frequency (LF), whereas driven measures were collected through squat‐stand maneuvers at point‐estimates of 0.05 and 0.10 Hz

The MCA and PCA spontaneous data produced average coherence values of 0.28–0.54 within the VLF range and 0.56–0.83 within the LF range (Figure 2), whereas at both driven frequencies (0.05 and 0.10 Hz), all coherence values were >0.95 across the cardiac cycle (Figure 3). Within the VLF and LF bands, no differences were found in coherence, (all *p* > .207), phase (all *p* > .290), absolute gain (all *p* > .291), or normalized gain (all *p* > .492) between any time points, in both the MCA and PCA across the cardiac cycle (Figure 2). Furthermore, no differences were found between any time points within the day in coherence, (all *p* > .273), phase (all *p* > .216), or normalized gain (all *p* > .084) while there was a trend for alterations in absolute gain (all *p* > .051) across the cardiac cycle in both the MCA and PCA for driven measures at 0.05 and 0.10 Hz (Figure 3). The spontaneous metrics across the cardiac cycle in both vessels produced a CoV ranging from 9.2% to 58%, whereas the driven CoV ranged from 0.2% to 21% (Figures 4 and 5).

### 3.4 Variations in autoregulation between sexes and vessels

**FIGURE 6:**
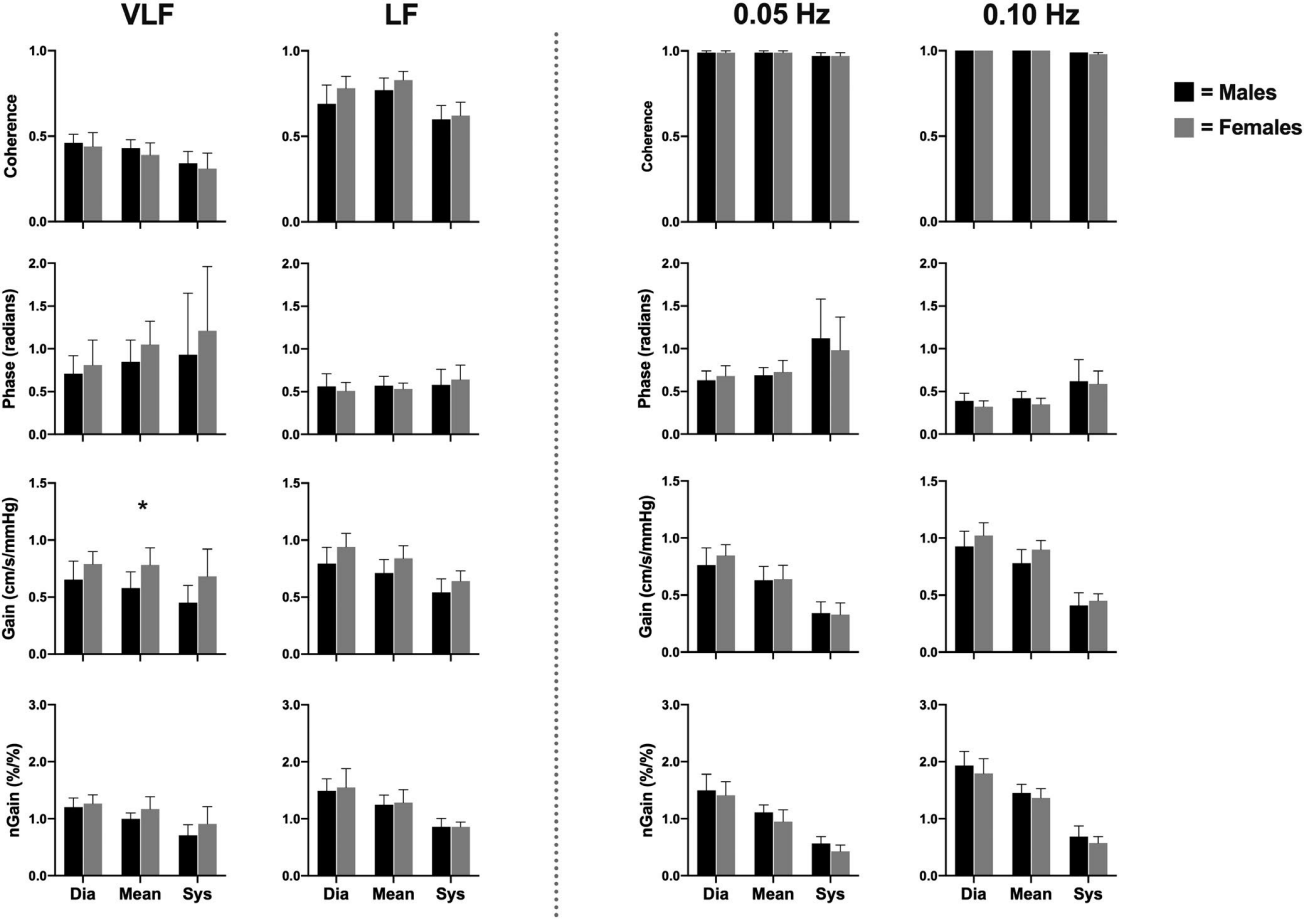
Mean with *SD* (error bars) of coherence, phase, gain, and normalized gain values within the middle cerebral artery across the cardiac cycle comparing males (*n* = 8) to females (*n* = 8). Spontaneous measures were collected as band averages within the very low frequency (VLF) and low frequency (LF), whereas driven measures were collected through squat‐stand maneuvers at point‐estimates of 0.05 and 0.10 Hz. The asterisk (*) represents a difference between sexes (*p* < .05). Males and females are represented by the black and gray bars, respectively

**FIGURE 7:**
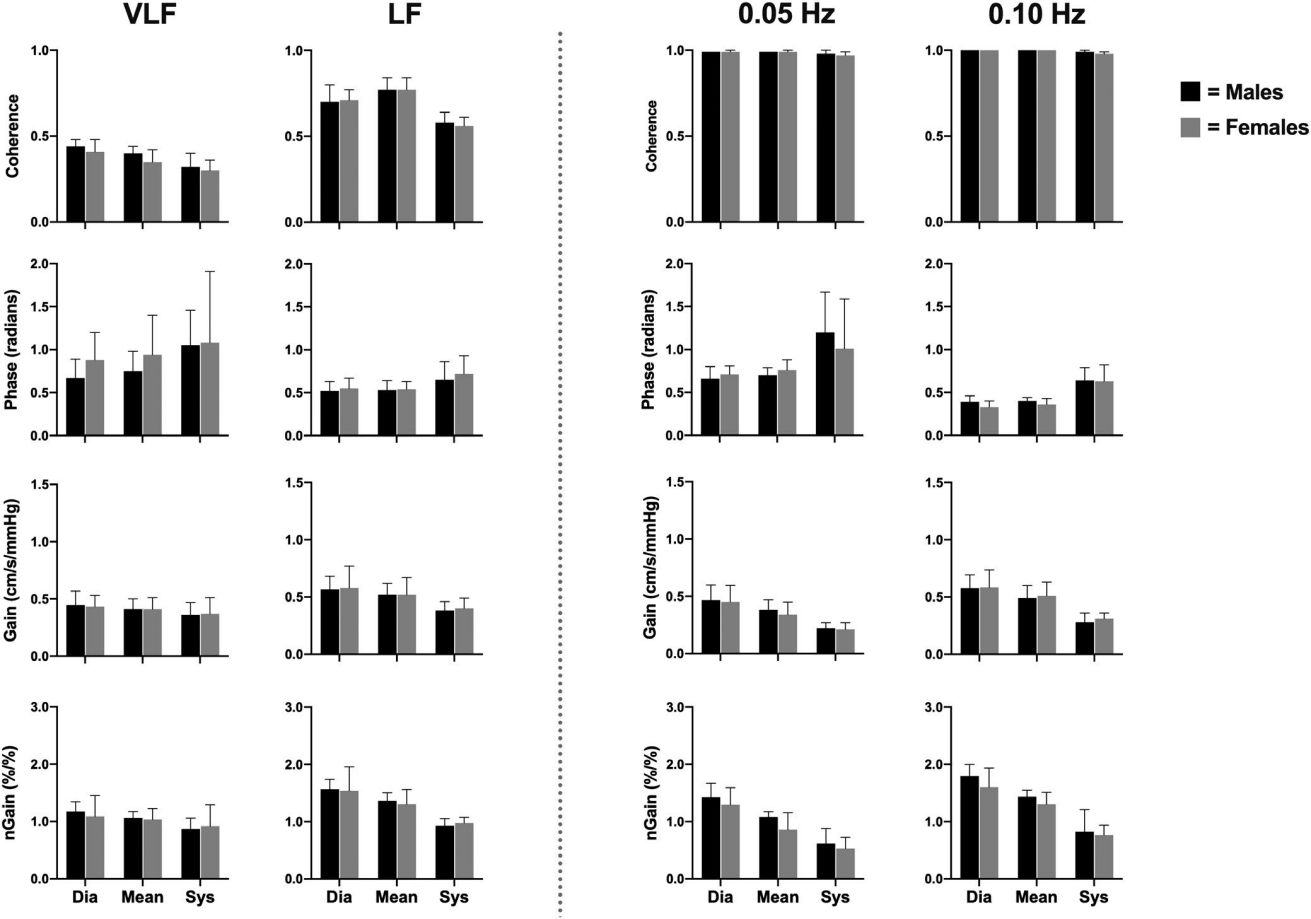
Mean with *SD* (error bars) of coherence, phase, gain, and normalized gain values within the posterior cerebral artery across the cardiac cycle comparing males (*n* = 8) to females (*n* = 8). Spontaneous measures were collected as band averages within the very low frequency (VLF) and low frequency (LF), whereas, driven measures were collected through squat‐stand maneuvers at point‐estimates of 0.05 and 0.10 Hz. Males and females are represented by the black and gray bars, respectively

**FIGURE 8:**
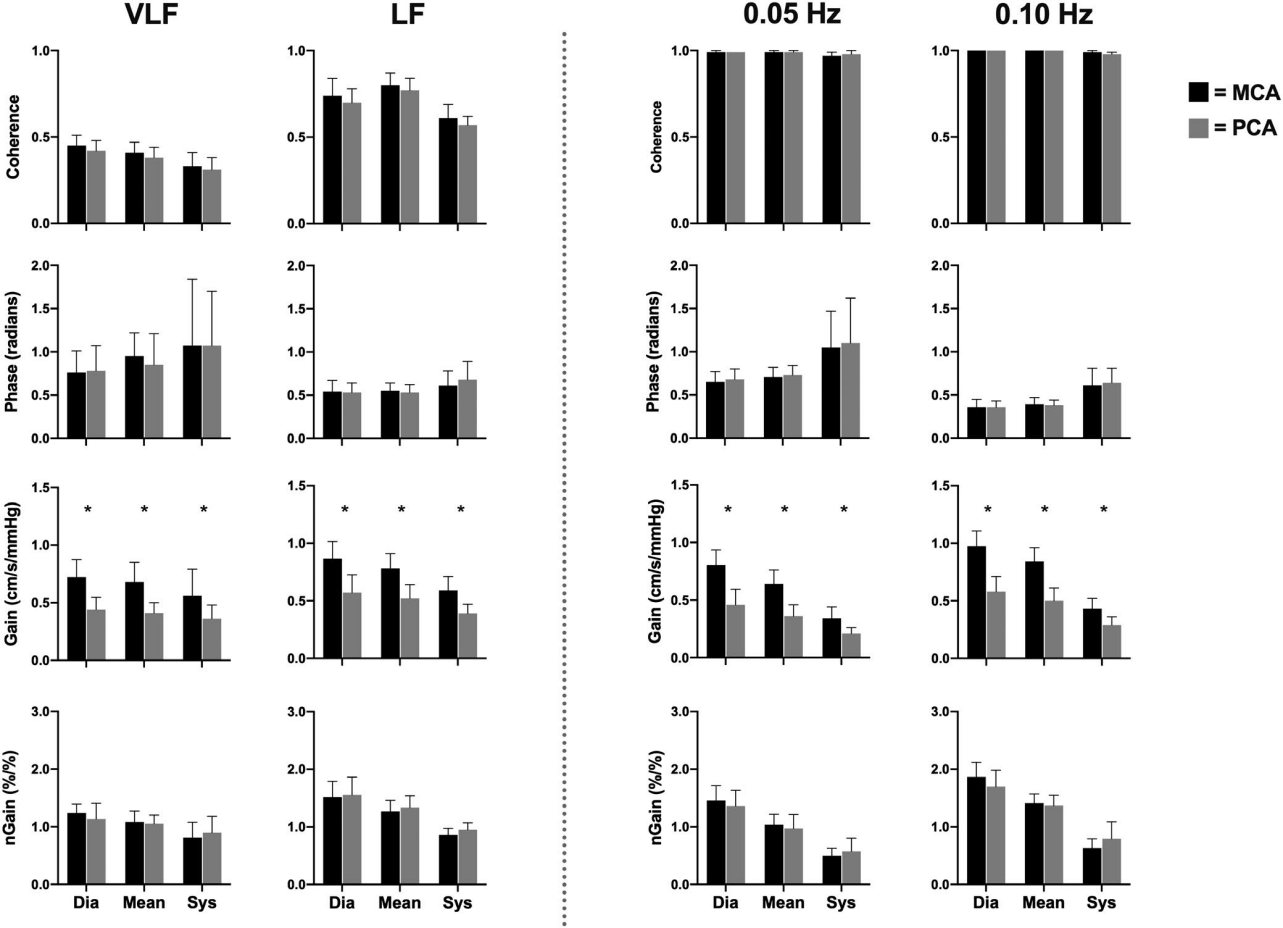
Mean with *SD* (error bars) of coherence, phase, gain, and normalized gain spontaneous and driven values across the cardiac cycle comparing the middle cerebral artery (MCA) to the posterior cerebral artery (PCA; *n* = 16). Spontaneous measures were collected as band averages within the very low frequency (VLF) and low frequency (LF), whereas driven measures were collected through squat‐stand maneuvers at point‐estimates of 0.05 and 0.10 Hz. The asterisk (*) represents a difference between vessels (*p* < .05). The MCA and PCA are represented by the black and gray bars, respectively

Within the spontaneous data, the mean VLF MCA absolute gain was lower in males compared to females (*p* = .036); however, no other VLF or LF spontaneous metric (coherence, phase, absolute gain, and normalized gain) differed between sexes (all *p* > .105; Figure 6). Furthermore, none of the aforementioned variables differed between males and females within driven frequencies (all *p* > .096; Figure 7). Coherence, phase, and normalized gain did not vary between the MCA and PCA in both spontaneous (all *p* > .099) and driven measures (all *p* > .178; Figure 8). Contrarily, all absolute gain measures were lower in the PCA compared to the MCA in both spontaneous (all *p* < .014) and driven metrics (all *p* < .001; Figure 8).

## 4 DISCUSSION

This study was the first to examine the reproducibility of both driven and spontaneous dCA measures across a single‐day while also presenting data between biological sexes and within the anterior and posterior cerebral circulation regions using squat‐stand maneuvers. The key findings from the investigation were fourfold: (a) Driven dCA measures demonstrated reduced levels of variation and greater reproducibility as demonstrated by the augmented coherence and minimal within‐subject CoV. (b) There was minimal variation present across the day due to diurnal factors, as noted in the driven oscillations across the cardiac cycle, whereas greater variation was observed within spontaneous measures. (c) The main conduit vessels within the cerebrovasculature (MCA and PCA) were regulated similarly within the squat‐stand protocols aside from the absolute gain metric. (d) No differences emerged between sexes in driven measures. Collectively, these results demonstrate driven dCA measures through squat‐stand maneuvers enable more robust interpretations to be drawn in anterior and posterior cerebral circulations across the typical workday (8:00–18:00) at all three phases of the cardiac cycle in both biological sexes. Furthermore, these results highlight the importance of normalizing the TFA gain value, as it is highly dependent upon the amount of blood pressure driving cerebral blood flow to the cerebrovascular region of interest.

### 4.1 Comparison with previous studies

Birch et al. (1995) were the first to propose squat‐stand maneuvers as a way to assess dCA; however, Claassen et al., (2009) were the first group to demonstrate that these maneuvers improved TFA coherence values, maximizing the signal‐to‐noise ratio, and reduced inter‐individual variability. These authors noted that squat‐stands maneuvers elicit higher reproducibility demonstrated by coherence values of 0.8, 0.9, and 0.9 at 0.025, 0.05, and 0.10 Hz, compared to spontaneous values of 0.4, 0.7, and 0.5, respectively (Claassen et al., 2009). A slight variation within the aforementioned study (Claassen et al., 2009) and this current investigation is attributable to the fact that they used shortened bands around the frequency of interest (0.025 Hz range was: 0.02–0.06 Hz; 0.05 Hz range was: 0.03–0.08 Hz; and 0.10 Hz range was 0.08–0.14 Hz), rather than using point‐estimates as done in this study and others (Smirl, Tzeng, et al., 2014; Smirl, Haykowsky, et al., 2014; Smirl, Lucas, et al., 2014; Smirl et al., 2015, 2016, 2018; Kostoglou et al., 2016; Wright et al., 2018, 2018a, 2018b). The latter technique augments that the TFA coherence values are consistent with the TFA coherence levels within the current investigation (all were >0.95: Figure 3). Furthermore, Smirl et al. (2015) found that an analogous verdict where driven oscillations lead to amplified reproducibility in TFA measures between 2 days in both frequencies of interest within the VLF and LF ranges (CoV < 20%). The coherence values within the driven blood pressure oscillations were 0.99–1.00 at both frequencies, opposed to the spontaneous of 0.44 and 0.69 in the VLF and LF, respectively. More so, the results within the current investigation are in agreement with these previous reports as all TFA coherence averages were >0.98 or greater in both vessels within diastolic and mean components and >0.95 in systolic (Figure 3). Additionally, the TFA measures during the spontaneous oscillations in blood pressure had less robust coherence values, which ranged from 0.28 to 0.54 within the VLF range and 0.56 to 0.83 in the LF range (Figure 2). Zhang et al., (1998) proposed a low coherence value which is thought to stem due to one or a combination of reasons including (a) there is no relationship between the input and output; (b) there is more than one input influencing the output; (c) the input‐output system is not linear; and/or (d) extraneous noise is causing measurement error. Of the four aforementioned reasons, only the fourth is capable to be manipulated using various methodological techniques. Therefore, the results in this investigation are consistent with the previous research which demonstrates that squat‐stands maneuvers are capable of enhancing the signal‐to‐noise ratio and thus reducing the likelihood of extraneous noise confounds the cerebral pressure–flow relationship compared to spontaneous measures. This is the primary reason which explains the reduced variability within the measures across the day and is aligned with the augmented coherence values (Figures 2 and 3). Ultimately, this means that the point‐estimates for the outcome measures of dCA (i.e., phase, absolute gain, and normalized gain sampled at the frequency of interest) are more likely to provide more accurate representations of the true estimate. To this point, the measures associated with the spontaneous oscillations had greater variation compared to driven oscillations as the associated TFA measures demonstrated CoV of 9.2%–58% and 0.2%–21%, respectively (Figures 4 and 5). The underlying physiological explanation for the elevated coherence is a body weight squat is known to engage skeletal muscles within the lower extremities, which subsequently results in chain reaction of: engagement of the skeletal muscle pump, elevated venous returns, greater cardiac outputs, and oscillations of ~30–50 mmHg in mean arterial pressure (Smirl et al., 2015). This was additionally detailed in the results section, where the blood pressure power spectrum densities were substantially enhanced while no differences were found between sexes. Conclusively, future studies employing repeated spontaneous measures should implement caution when interpreting the results.

Moreover, prior to this study, there have been no studies that have examined the effect diurnal variation on driven dCA measures across the workday. The current findings demonstrate that driven TFA coherence, phase, absolute gain, and normalized gain were consistent at both frequencies within both conduit vessels (Figure 3) and were reduced within‐subject variation across the day compared to spontaneous (Figures 4 and 5). Finally, it is important to note that within both spontaneous and driven measures, systolic coherence was slightly lower relative to the mean and diastolic components of the cardiac cycle. This is homogeneous with previous research (Smirl et al., 2018), which revealed systole to have a larger phase shift and a reduced normalized gain, emblematic that more protective buffering is present with respect to the systolic aspect of the cardiac cycle. Henceforth, this explains why more variability was found within the systolic measures compared to mean and diastolic (Figures 2, 3, 4, 5). Furthermore, driven phase and normalized gain metrics shown in Figures 6, 7, 8 clearly demonstrates that consistency with the prior literature as these phenomena demonstrate that phase is similar between mean and diastole, but greater in systole, whereas within the normalized gain, the systolic values are lower than the other two cardiac cycle components (Smirl et al., 2018; Wright et al., 2018). Disparagingly to phase, however, mean normalized gain was different than diastole, with the latter having the highest amplitude in driven measures (Figures 6, 7, 8).

### 4.2 Diurnal variation in dCA measures across the workday

There is substantial literature describing the circadian variation in CBV (Conroy, Spielman, & Scott, 2005) and blood pressure independently (Degaute, Van De Borne, Linkowski, & Van Cauter, 1991; Kawano, 2011). However, there are considerable discrepancies within the previous investigations. While the majority of the studies have noted CBV to be the lowest during sleep and in the early morning compared to the afternoon or evening (Conroy et al., 2005; Droste, Berger, Schuler, & Krauss, 1993; Hajak et al., 1994; Sakai, Meyer, Karacan, Yamaguchi, & Yamamoto, 1979). There have been a few studies that have noted the opposite (Hossmann, Fitzgerald, & Dollery, 1980; Jiang et al., 2016). A recent study noted that using magnetic resonance imaging decreased neural activation and blood‐oxygen‐level‐dependent signal in the medial temporal and occipital lobes in the afternoon (compared with morning), while the opposite effects were observed in the prefrontal cortex within the same investigation (Jiang et al., 2016). Additionally, it has been speculated that reductions in cerebral blood flow at night may result in an elevated risk for a potential ischemic stroke (Hossmann et al., 1980). Furthermore, blood pressure has been shown to rise rapidly upon waking, plateau during the morning, decline in the early afternoon, and rise again in the early evening before decreasing while one sleeps (Degaute et al., 1991; Kawano, 2011). As dCA is underpinned by the relationship between arterial blood pressure and CBV, it would make intuitive sense dCA would demonstrate similar diurnal variation across the 24‐hr day. To date, only one prior study has examined this relationship, finding that dCA was impaired in the morning (06:00–08:00) compared to results from the previous evening (18:00–20:00; Ainslie, Murrell, et al., 2007). These results were attributed to a reduction in cortical oxyhemoglobin concentration and MCA blood velocity (Ainslie, Murrell, et al., 2007). A limitation of this previous study, however, was that the dCA response was only examined at these two specific time points and not continuously across the evening, during which time the probes were removed while participants slept and reinstrumented the following morning. To address this limitation, the current investigation performed serial follow‐ups at seven time points throughout the day while maintaining the probe placements throughout the day and observed there was minimal variation from 08:00 to 18:00. Ultimately, this eliminated the possibility that the results were impacted, due to the TCD probe insonating the vasculature at a slightly different angle. The conflicting findings between the two studies can also potentially be attributed to how the investigations were performed. Ainslie, Barach, et al. (2007) examined this 30 min upon waking, as this time has shown to have a reduction in endothelial function which would reduce the dCA response while their latter time point would have been more consistent with the current study, where the first measure was performed more than an hour upon waking, likely where endothelial function returned to normal daily functioning. The physiological underpinnings of the maintained dCA across the day may be the resultant that this process is pertinent in regulating cerebral blood flow to ensure there is not under‐ or over‐perfusion, which would potentially lead cerebrovascular accidents (Jordan & Powers, 2012). Moreover, an increased likelihood of having a cerebrovascular incident has been demonstrated to occur immediately upon waking, which may be an additional consideration for Ainslie and colleagues to have observed a reduced dCA in the morning from 6:00 to 8:00 a.m. This could potentially be related to a surge in cortisol, which is known to peak around 8:30 a.m. (Chan & Debono, 2010); however, further research is needed to confirm the association with this potential mechanism.

Furthermore, variation in both spontaneous and driven measures was examined, with driven blood pressure oscillations showing less variance and more consistency across the workday (Figures 2, 3, 4, 5). The underlying rationale for this within driven measures is the augmented hemodynamics in dCA measures, shown by the average 59‐fold and 41‐fold increase in power spectrum densities at point‐estimates 0.05 and 0.10 Hz, respectively. As previously mentioned, physiologically, the squat‐stand maneuvers produce blood pressure oscillations which can range ~30–50 mmHg. This orthostatic challenge occurs because as an individual squats down, they engage the skeletal muscle pumps in the legs, resulting in an upswing in venous return to the heart (Smirl et al., 2015). This in turn produces an elevation in cardiac output and a short‐term surge in blood pressure, which peaks roughly 5–7 s following the squat (Aaslid et al., 1989). The decline in blood pressure upon standing can be attributed to the relaxation of the leg skeletal muscle pumps and a reduced cardiac output, enabling blood to pool within the venous aspect of the lower body vasculature (Wieling, Krediet, Van Dijk, Linzer, & Tschakovsky, 2007). As such, these maneuvers greatly enhance the power of the blood pressure signal (input) which is transmitted to the brain and augments the power of the CBV signal (output), this greatly enhances the signal‐to‐noise ratio for the TFA analysis, improving coherence to near‐linear levels (>0.99; Smirl et al., 2015). The improved reproducibility of the associated phase and normalized gain measures enhances the interpretability of these metrics (Simpson & Claassen, 2018; Tzeng & Panerai, 2018) and our current results reveal examining the linearity of dynamic dCA measures using driven measures can test for reproducible measures anywhere across the typical workday (8:00 a.m. to 7:00 p.m.).

### 4.3 Sex differences in dCA measures

Several studies have investigated if sex‐related differences are present for dCA metrics in: children (ages: 4–8; Tontisirin et al., 2007), adolescents (ages: 10–16; Vavilala et al., 2005), adults (Deegan et al., 2010; Favre & Serrador, 2019; Labrecque et al., 2019; Wang et al., 2005), and the elderly (Deegan, Sorond, Lipsitz, Ólaighin, & Serrador, 2009). Females have generally reported a higher MCA blood velocity compared to males, which is thought to stem from several mechanisms including: (a) greater estrogen levels and its vasodilatory effect through the elevation of endothelial nitric oxide synthase (Shamma, Fayad, Brass, & Sarrel, 1992); (b) an elevated concentration of cyclooxygenase (Peltonen et al., 2016); and (c) contribution of progestin (Krause, Duckles, & Pelligrino, 2006). Nonetheless, the overall findings from these investigations have elicited conflicting findings. Vavilala et al. (2005) noted that there was a higher MCA autoregulatory index (ARI) in males, and contrastingly noted a higher basilar artery ARI in females. They speculated that these differences in the posterior cerebrovasculature may be related to boys being at a higher (although uncommon) risk of strokes in the posterior circulatory bed. Conversely, Tontisirin et al., (2007) observed no differences in dCA between sexes in a slightly younger cohort of prepubescent children (age 4–8 years). In the adult‐based literature, Wang et al. (2005) reported higher TFA coherence and gain measures within the VLF and HF bands in women during spontaneous supine oscillations, while men had a higher LF coherence during upright tilt. In a similar population, Deegan et al., (2010) noted women had higher ARI values than males but only in the anterior cerebral artery (no differences present in the MCA) when the subjects were in a supine or seated position. They accredited these differences may be related to the females in their study having blunted P_ET_CO_2_ levels which differentially affected the anterior and middle cerebral circulatory beds. Finally, in the elderly subjects (mean age 78 years) investigated by Deegan et al., (2009), it was observed that females had higher ARI values while males demonstrated higher VLF and cardiac frequency absolute gain values. These differences were speculated to indicate elderly females have more reactive cerebral vessels which may have some neuroprotective effects as noted by the improved dCA metrics (Deegan et al., 2009). While previous literature has noted numerous potential dCA differences with respect to biological sex, the current investigation was unable to confirm the previous findings as there were no sex differences observed between young healthy adults in either the MCA and PCA (Figures 6 and 7). The convergent findings may be due to the difference in methodology employed between the various investigations where this study used squat‐stand maneuvers and assessed dCA through TFA. Conversely, the previously mentioned studies assessed dCA via spontaneous blood pressure oscillations or other methods (i.e., thigh cuff inflation/deflation, supine‐to‐sitting, sit‐to‐stand, or ARI). The vast majority of the previous research has focused on either ARI or employing spontaneous oscillations for TFA based assessments. Nonetheless, two previous studies have also examined sex differences in dCA within adults, utilizing squat‐stand maneuvers; however, this investigation differs from these slightly. Favre et al. (2019) had both male and female participants perform squats at a frequency of 0.05 Hz only and found that females had lower MCA gain and higher MCA phase values. Furthermore, the coherence values within the MCA was lower in females (~0.92) compared to males (~0.96) during the squat‐stands. Moreover, Labrecque et al. (2019) demonstrated females had a 48% greater gain metric compared to men during driven blood pressure oscillations at 0.05 Hz, with no differences occurring at 0.10 Hz. Similarly, these authors found lower coherence values within female participants (~0.90) compared to males (~0.99). The results within the current investigation contradict the findings within the two preceding studies, where no driven measures were found to be different between biological sexes (Figures 6 and 7). This could potentially stem from three potential reasons. First, there were no differences within TFA coherence values between males and females (all *p* > .504) within all driven measures in the current investigation. Second, this may be attributable to the notion dCA metrics were collapsed across seven time points for each participant, thus increasing the accuracy of each estimate. Third, several research groups have noted that cardiorespiratory fitness levels may play a factor in influencing dCA metrics (Drapeau et al., 2019; Labrecque et al., 2017; Lind‐Holst et al., 2011). Although each individual's maximal oxygen uptake was not precisely determined, recreationally active healthy young adults were sampled within the current investigation thus would be expected to have relatively average oxygen uptake levels. Opposingly, the abovementioned studies that assessed fitness on dCA through squat‐stand maneuvers (Drapeau et al., 2019; Labrecque et al., 2017) used endurance athletes, who would be expected to have a much higher maximal oxygen uptake, and thus may potentially partially explain the discrepancy in results. Nevertheless, based on the results within this investigation, it appears both biological sexes autoregulate their brain blood flow in a harmonious manner throughout a given day.

### 4.4 Differences in regulation between vessels

Park et al. (2003) found that the autoregulation index did not differ within the MCA and the basilar artery in young healthy participants (27 ± 9 years, range: 19–46), which is similar to the age range in this study (25 ± 4 years, range: 21–37). Similarly, Tontisirin et al. (2007) found that children (age range 4–8) had higher middle and basilar blood velocities, but there were no differences in ARI when changing position from supine to sitting 90°. Furthermore, these authors examined both the posterior and anterior circulations finding they were regulated similarly (MCA ARI = 0.96 ± 0.09; Basilar ARI = 0.94 ± 0.12). In contrast, Vavilala et al., (2005) revealed that in healthy adolescents (age range: 10–16 years), females had greater dCA in the basilar artery, whereas it was enhanced for females in the MCA. These results demonstrate a difference in autoregulation between the posterior and anterior circulations; however, their study assessed static cerebral autoregulation in a similar supine to sitting 90° position change. Finally, Haubrich et al. (2004) found the TFA phase shift to be relatively similar within the MCA and PCA; however, these authors found absolute gain increases ranging from 28% to 53% within the PCA relative to the MCA gain. However, within this study, absolute MCA gain was found to be higher than the PCA ranging from 33% to 40% in spontaneous and 32% to 43% in driven; which similarly to the discrepancy found within sex differences is likely attributable to the different methodology employed to index dCA. Smirl, Lucas, et al. (2014) congruously found that the MCA absolute gain was higher than in the PCA, when assessing CA using squat‐stand maneuvers. This divergence in absolute gain metrics is likely the result of 72% of cerebral blood flow being directed to the carotid artery, compared to 28% within the vertebral circulation (Zarrinkoob et al., 2015). The divergence in blood flow distribution highlights the results seen in Figure 8, and showcases the importance of normalizing gain metrics in future dCA investigations when trying to understand dCA within different circulatory beds. Nonetheless, this investigation and the aforementioned study by Haubrich et al., (2004) found that the TFA phase shift was analogous between vessels.

### 4.5 Implications for future assessments of dCA

From this investigation, several implications can be drawn, which are imperative for future studies examining dCA metrics. The results within this study are in agreement with previous research (Claassen et al., 2009; Smirl et al., 2015; Smirl, Lucas, et al., 2014) demonstrating driven oscillations produce more robust measures as demonstrated by the greater coherence values (>0.95) and a lower CoV (<21%) than spontaneous measures (VLF: 0.28–0.54 and <58%; LF: 0.56–0.83 and <41%), respectively. Moreover, this investigation found minimal differences in driven measures across the typical workday, whereas spontaneous metrics were much more variable. The latter results are likely attributed to measurement error rather than diurnal variation, and thus future studies using squat‐stand maneuvers can index the cerebral pressure–flow relationship at any time point across the workday with highly reproducible results. Therefore, when feasible, squat‐stand maneuvers should be utilized to quantify dCA. Nonetheless, in clinical populations that are unable to perform these maneuvers (i.e., spinal cord injury, severe brain injury, etc.; Phillips, Krassioukov, Ainslie, & Warburton, 2014), other driven techniques should be used over spontaneous measures as the latter methodological approach is confounded by the influence of physiological “noise” which can result in measurement errors occurring (Smirl et al., 2015). Moreover, this investigation only examined spontaneous and driven dCA metrics at rest and during squat‐stand maneuvers within healthy individuals and thus future research is warranted to examine if the same findings occur during the employed methodologies when various physiological perturbations (e.g., carbon dioxide, medications, etc.) are added as confounders.

Conclusively, the current results demonstrate that the two main conduit vessels within the brain regulate analogously when dCA data are normalized and there were no biological sex differences noted between young healthy recreationally active males and females when females were tested within the early follicular phase (days 3–7).

### 4.6 Limitations

A commonly known drawback of using transcranial Doppler ultrasound to quantify dCA is due to the fact it is only capable of capturing velocity, rather than providing a true metric of cerebral blood flow. Nonetheless, using the assumption that velocity is equivocal to flow and with the notion the vessel being insonated remains constant, one is theoretically able to calculate CBV. Moreover, a recent study used high‐resolution magnetic resonance imaging to assess if the vasculature remains constant across various P_ET_CO_2_ levels (Coverdale, Gati, Opalevych, Perrotta, & Shoemaker, 2014; Verbree et al., 2014). It was found the diameter remains relatively constant when P_ET_CO_2_ levels remain within 8 mmHg of eucapnia (Ainslie & Hoiland, 2014). All squat‐stand protocols were performed at eucapnia within this investigation and thus the data collected are likely to accurately portray a surrogate of cerebral blood flow. Moreover, it is important to note that blood pressure oscillation directionality was not examined within the current investigation, as previous research has demonstrated the presence of hysteresis within driven oscillations, and thus future research in this realm is warranted (Brassard et al., 2017; Panerai et al., 2018). Finally, the participants within this study were recreationally active, healthy university students and therefore the results may not have great external validity to elderly, clinical, or trained populations.

### 4.7 Conclusions

In summary, driven dynamic dCA metrics elicited more robust measures compared to spontaneous metrics across the day (8:00–18:00). Additionally, both MCA and PCA regulated similarly across the day, as well did both males and female participants. Therefore, in the future when feasible, squat‐stand maneuvers should be used to assess dCA in participants, which can be performed in both vessels, across the cardiac cycle and in both biological sexes, as there is minimal influence of diurnal variation across the day within these measures. However, as mentioned, in some clinical populations this technique may not be possible, and thus other driven techniques should be used over spontaneous measures. Finally, future studies assessing dCA using repeated spontaneous measures should use caution when interpreting the results, as this study demonstrated TFA variables to vary substantially across the day.

## CONFLICT OF INTEREST

The authors declare they have no conflicts of interest.

## AUTHOR CONTRIBUTIONS

J.S.B. and J.D.S. designed the study. J.S.B., P.C., A.M., O.M., and J.D.S. performed data collection. J.S.B. and J.D.S. performed statistical analysis and interpreted the data. J.S.B., P.C., A.M., O.M., and J.D.S. contributed to writing and proofing the final version of the manuscript.
